# Discoveries beyond *BRCA1/2*: Multigene testing in an Asian multi-ethnic cohort suspected of hereditary breast cancer syndrome in the real world

**DOI:** 10.1371/journal.pone.0213746

**Published:** 2019-03-15

**Authors:** Samuel Guan Wei Ow, Pei Yi Ong, Soo-Chin Lee

**Affiliations:** 1 Department of Hematology-Oncology, National University Cancer Institute, Singapore, Singapore; 2 Cancer Science Institute, National University of Singapore, Singapore, Singapore; Ohio State University Wexner Medical Center, UNITED STATES

## Abstract

**Background:**

Due to historically low uptake of genetic testing, the mutational spectrum of Asians with Hereditary Breast Cancer (HBC) is not well understood. This study sought to understand the incidence and spectrum of germline mutations in Asian patients with suspected HBC in a clinic setting.

**Methods:**

1056 patients with suspected HBC were seen in our Cancer (CA) Genetics Clinic from 2000–2017, of which 460 underwent genetic testing.

**Results:**

Of 460 probands tested, 93% were female, 61% Chinese, 90% had prior CA, with 19% (77/414) having ≥2 primary CA. Median age at CA-diagnosis was 43y (17–83); 70% had Breast CA (BC) and 25% Ovarian CA (OC). 34% had young-onset BC, 8% bilateral BC, and 4% BC/OC. Majority had family history of BC (53%) or OC (20%). 57% underwent multigene testing (14–49 genes), 34% targeted testing, and 8% predictive testing. 30% were found to have a pathogenic mutation: 80% in *BRCA1/2* (8 novel mutations noted). Of 33 non-*BRCA1/2* pathogenic mutations detected, 61% were in 11 BC genes while 39% were in non-BC genes suggestive of alternative CA syndromes. Testing beyond *BRCA1/2* impacted management for 15.9% (22/138) of carriers, but extensive testing identified variants of uncertain significance (VUS) in up to 44.5% of probands. Restricting multigene panel testing to a guideline-based 20-gene panel including Lynch Syndrome genes was found to be most optimal, detecting 94.6% of mutation carriers while reducing VUS rate to 21.5%.

**Conclusions:**

Evolution of CA Genetics testing strategy to a multigene approach facilitated detection of pathogenic mutations in non-*BRCA1/2* genes and aided management. Guideline-based panel testing is feasible and can be offered in Asians with suspected HBC.

## Introduction

Much progress has been made in the diagnosis and management of hereditary breast cancer syndrome in the past decades. Previously thought to be restricted to the *BRCA1/2* genes, astute clinical observation and family-based studies have led to the discovery of other high penetrance breast cancer predisposition genes such as *TP53* (Li Fraumeni syndrome) and *PTEN* (Cowden Syndromes).[1, 2] With further advancements in genetic testing capability, particularly with the use of high throughput technology such as next-generation sequencing, moderate-penetrance breast cancer genes such as *PALB2*, *CHEK2* and *ATM* have been uncovered, and clinicians are beginning to gain deeper understanding of the clinical phenotype of breast cancer families harboring defects in these genes.[3]

Cancer genetic services remain the main provider of genetic cancer risk assessment and serve as dedicated resources for probands in the cancer prevention and treatment continuum, from pre-test counselling, formulating appropriate test strategy, post-test counselling, to recommending personalized cancer surveillance and management, and facilitating predictive testing for kindred. The goal of genetic cancer risk assessment is to help identify individuals and families who have a genetic predilection to cancer, and recommend preventative or screening measures to reduce their risk.[4] The discovery of *PARP* inhibitors as effective treatment for *BRCA1/2*-associated breast and ovarian cancers has also augmented the role of genetic cancer risk assessment to advise mutation positive patients on specific cancer therapy.[5, 6]

While cancer genetic services have been present in Asia since the 2000s, the uptake for germline genetic testing in the clinic has been initially low largely due to cost concerns.[7] As such, much of the literature and guidelines for hereditary breast cancer have been driven by major cancer centers in the West.[8] However, small studies from Asian groups have reported novel mutations in breast cancer patients from this part of the world.[9, 10] The use of multigene panel testing in the clinic has also opened the door to discovering non-*BRCA1/2* genetic mutations, allowing insight to the mutational spectrum in this still poorly understood population. We describe the outcomes of clinical genetic testing including the use of multigene panels in a multi-ethnic cohort at a tertiary cancer center in Asia.

## Materials and methods

Clinical and genetic information were collected with approval from the National Healthcare Group Doman Specific Review Board (DSRB 2000/00511). All procedures performed in studies involving human participants were in accordance with the ethical standards of the institutional and/or national research committee and with the 1964 Helsinki declaration and its later amendments or comparable ethical standards. Written informed consent was obtained from all individual participants included in the study.

### Clinical cancer genetics service

The Clinical Cancer Genetics Service at the National University Cancer Institute, Singapore was established in 2001, and serves as a major referral center for genetic cancer risk assessment in Southeast Asia and the Middle East.[11] Three-generation family cancer pedigrees were constructed and evaluated for each proband by a cancer genetics counselor, with further counseling and assessment in conjunction with a medical oncologist specialized in cancer genetics.

Genetic testing was offered to probands with 5% or greater putative chance of having an underlying genetic predisposition, as determined by prevailing guidelines from organizations including, but not limited to, the National Comprehensive Cancer Network, Society of Gynecologic Oncology and United States Preventive Services Task Force, and supplemented by the use of clinically relevant prediction models such as Couch Model and Penn II Model.[12] In the initial period, single syndrome-based testing of the cancer predisposition gene(s) felt most likely to be implicated was performed, using Sanger sequencing with or without deletion/duplication analysis. In probands with a family history of a known germline mutation, predictive testing of the said mutation was performed (single site mutation analysis). Multigene panel testing with next-generation sequencing was subsequently offered since 2014, when clinical grade next-generation sequencing (with screening of large deletions, duplications and rearrangements using laboratory-specific protocols such as multiplex ligation-dependent probe amplification, multiplex quantitative polymerase chain reaction, microarray-comparative genomic hybridization, read-depth and split-read analysis) came into widespread use. All samples for clinical genetic testing were analyzed by commercial laboratories certified by regulatory bodies such as the College of American Pathologists, and United Kingdom Accreditation Service. Genetic test results were extracted from formal genetic test reports, with variant calling and classification made based on their databases and algorithms employed by the respective commercial laboratories. The number of genes included in the panels expanded with time (range 14, 49) and are listed in S1 Table.

### Statistical methods

Patient characteristics and gene test results were tabulated and described. Clinical characteristics and test results were compared using chi-square tests (for proportions of categorical variables) and student’s t-test (for means of continuous variables). Logistic regression was used to calculate odd ratios for binary variables. All tests were 2-sided with significance level set at 0.05. Statistical analysis was performed using Intercooled Stata release 14. (StataCorp. 2015. Stata Statistical Software: Release 14. College Station, TX: StataCorp LP.)

## Results

### Patient characteristics

During the period spanning January 2001 to May 2017, 1056 patients with suspected hereditary breast cancer syndrome were reviewed at the Cancer Genetics Clinic. 94.9% were female, with majority being Chinese (63.3%) followed by Malay (9.8%), Indian (9.8%), and others (17.1%), including 2.7% Middle Eastern. Mean age at the time of visit was 47.2 years (range 12, 91). 17.1% were cancer-free. Of the 876 patients with prior diagnosis of cancer, 82.5% had one primary, 15.7% had two primary cancers, and 1.7% had three or more primary cancers. The mean age of first cancer diagnosis was 43.9 years (range 11, 87). The most common site of primary cancer was breast (75.9%), followed by ovary (17.8%) and prostate (1.5%). More than half of the patients were referred by medical oncologists (63.5%), with the remaining from surgeons (15.3%), gynecologists (9.6%), self (6.2%) and general practitioners (2.6%). Full details are presented in S2 Table.

Of the 1056 patients suspected to have hereditary breast cancer syndrome, 43.6% (460/1056) underwent genetic testing, including 41 individuals (4% of the entire cohort) who had a positive family history of a germline *BRCA1/2* mutation (Table 1). Three-fifths were of Chinese ethnicity, followed by Indians (10.7%), Caucasian (7.4%) and Malay (5.9%). 10.0% (46/460) were cancer-free. The mean age of cancer diagnosis was 44.4 years, with more than 90% having breast and/or ovarian cancer. A significant majority had a family history of breast cancer (52.6%), ovarian cancer (19.8%), or both (12.1%). 20.2% (93/460) were assessed to also have features suggestive of alternative hereditary cancer syndromes, most commonly Lynch Syndrome (65, 69.9%), Li Fraumeni (11, 11.8%) and Cowden Syndrome (11, 11.8%) which have overlapping phenotypes with *BRCA1/2* hereditary breast cancer syndrome.

**Table 1:** Clinical characteristics of probands who underwent genetic testing (N = 460).

Characteristics		No.	(%)
Gender	Female	429	(93.3)
	Male	31	(6.7)
Age at Time of Visit	Mean (Range)	47.8y	(18, 85)
	Median	46y	
Ethnicity	Chinese	279	(60.7)
	Indian	49	(10.7)
	Caucasian	34	(7.4)
	Malay	27	(5.9)
	Indonesian	26	(5.7)
	Middle Eastern	17	(3.7)
	Filipino	8	(1.7)
	Others^a^	20	(4.3)
Number of Cancer (CA) Primaries	Cancer-free	46	(10.0)
	1	337	(73.3)
	2	69	(15.0)
	≥3	8	(1.7)
Age of first CA Onset (n = 414)	Mean (Range)	44.4y	(17, 83)
	Median	43y	
Primary Site of CA (n = 414)	Breast	289	(69.8)
	Ovary	102	(24.6)
	Prostate	8	(1.9)
	Colon	5	(1.2)
	Endometrium	3	(0.7)
	Pancreas	3	(0.7)
	Adrenal	1	(0.2)
	Cervix^b^	1	(0.2)
	Paraganglioma^c^	1	(0.2)
	Thyroid	1	(0.2)
Personal History	Breast CA ≤40y	158	(34.4)
	Bilateral Breast CA	38	(8.3)
	Breast and Ovary CA	17	(3.7)
	Pancreas and/or Prostate CA	12	(2.6)
	Male Breast CA	7	(1.5)
Family History	Breast CA	242	(52.6)
	Ovary CA	91	(19.8)
	Prostate CA	31	(6.7)
	Pancreas CA	15	(3.3)
	*BRCA1/2* Mutation	41	(8.9)

^a^Eurasian (n = 4), Myanmese (n = 4), Vietnamese (n = 4), South American (n = 4), Cambodian (n = 1), Japanese (n = 1), Mauritian (n = 1), Thai (n = 1)

^b^Patient was diagnosed with metastatic cervical cancer, and noted to have a BRCA1 mutation on somatic tumor sequencing

^c^Patient has family history of young-onset breast cancer

### Germline genetic testing strategy and results

In the tested cohort of 460 probands, 57.2% (n = 263) underwent panel-based testing, 33.9% (n = 156) had targeted gene testing, while 8.1% (n = 41) underwent single site mutation analysis. Test details are highlighted in Table 2. 30.0% (138/460) of probands were found to have a pathogenic mutation on testing, while 42.8% did not have any mutations found. 27.2% were noted to have a variant of uncertain significance (VUS) in the absence of a pathogenic mutation.

**Table 2:** Test strategy (N = 460).

Strategy	Genes tested	No.	(%)
Predictive Testing (Single site mutation analysis) (n = 41)	*BRCA1*	24	(57.1)
	*BRCA2*	15	(35.7)
	*BRCA2* and *RET*	2	(4.8)
Targeted Testing (n = 156)	*BRCA1/2*	149	(95.5)
	*BRCA1/2* and Mismatch Repair Genes	6	(3.8)
	*BRCA1/2* and PTEN	1	(0.6)
Panel-based Testing (n = 263)	14-gene panel	2	(0.8)
	29-gene panel	32	(12.2)
	34-gene panel	83	(31.6)
	49-gene panel	146	(55.5)

#### Pathogenic mutations

**Fig 1:**
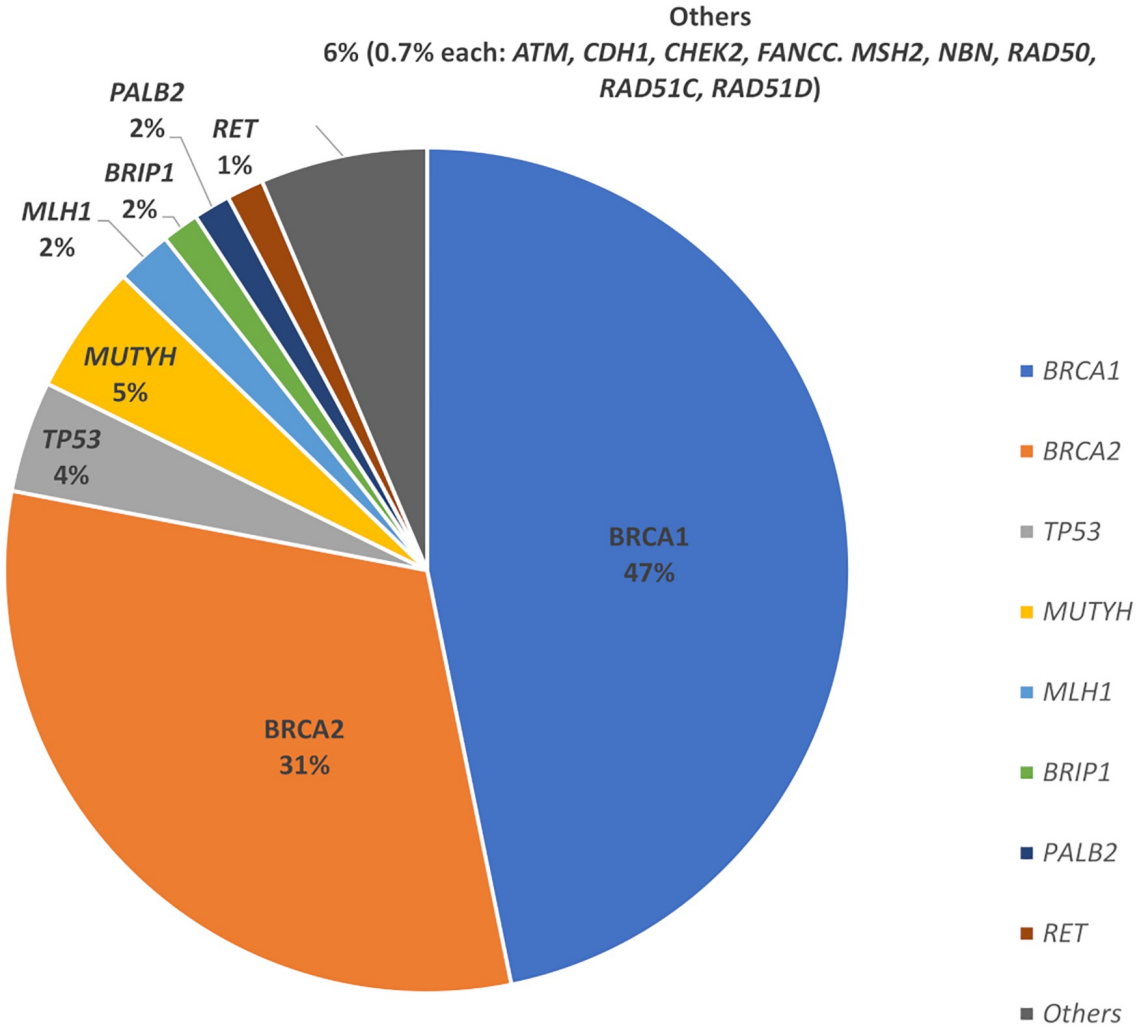
Proportion of pathogenic mutations detected in BRCA1/2 and non-BRCA1/2 genes.

Of the 138 probands found to have a pathogenic mutation, 110/138 (79.7%) were in *BRCA1/2* genes (66 in *BRCA1*, 44 in *BRCA2*), as indicated in Fig 1. A total of 69 unique mutations were seen in *BRCA1/2*, of which eight were novel mutations not previously described at the time of testing. Frameshift mutations were most common (63.0%), followed by nonsense mutations (27.3%) and large deletions (5.5%). Frameshift and nonsense mutations made up 95.5% of mutations in *BRCA2* compared to 80.3% of mutations in *BRCA1* (p<0.01). There were a larger variety of mutations seen in *BRCA1* compared to *BRCA2*, including large deletions (9.1%), intronic changes (3.0%), duplication mutations (3.0%), missense mutations (3.0%) and splice site mutations (1.5%). Of interest, we found one recurring mutation each in Chinese (*BRCA2*:*c*.*3109C>T* nonsense mutation in 3 unrelated individuals) and Malays (*BRCA1*:*c*.*2726dupA* frameshift mutation in 4 probands from 3 unrelated families). Detailed information on the pathogenic mutations found and the associated pedigree are provided in S3 Table.

32/138 (23.2%) mutation-positive patients had a total of 33 pathogenic mutations in non-*BRCA1/2* genes: *MUTYH* (mono-allelic; n = 7), *TP53* (n = 6), *MLH1* (n = 3), ATM (n = 2), *BRIP1* (n = 2), *FANCC* (n = 2), *PALB2* (n = 2), *RET* (n = 2), and 1 each in *CDH1*, *CHEK2*, *MSH2*, *NBN* and *RAD50/51C/51D*. 10 novel mutations were detected in 8 non-*BRCA1/2* genes (S3 Table). Among the 33 mutations seen in the non-*BRCA1/2* genes, 20/33 (60.6%) were in high and moderate-penetrance breast cancer genes. 13/33 (39.4%) were in four non-breast cancer genes, of which 4/13 were in *MLH1* and *MSH2* which are associated with Lynch Syndrome. 7/13 had pathogenic heterozygous mutations in *MUTYH* which are not thought to be causative of the probands’ cancers and therefore believed to be incidental findings with limited clinical relevance. Of interest, four individuals with *BRCA1/2* mutation had an incidental finding in another gene (two related individuals with *BRCA2* and *RET*, one with *BRCA2* and *MUTYH*, and one with *BRCA1* and *FANCC*) which did not correlate with the clinical presentation.

**Fig 2:**
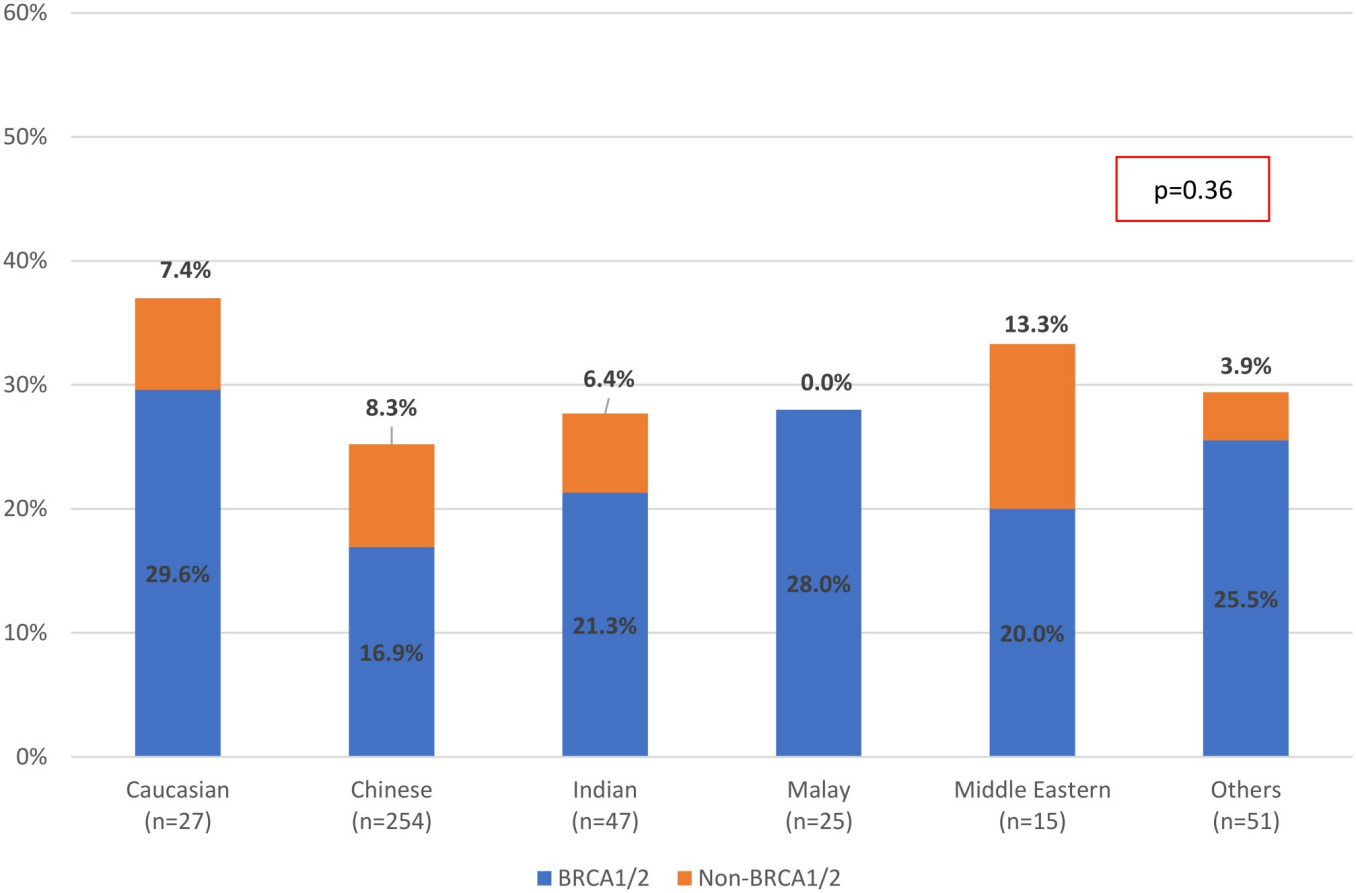
Proportion of pathogenic mutations detected in *BRCA1/2* and non-*BRCA1/2* genes by Ethnicity (N = 419). **(Excluding n = 41 probands who underwent predictive testing)** Proportion of Pathogenic Mutations by Ethnicity.

On multivariate analysis of possible risk factors, a personal history of more than one cancer, personal history of ovarian cancer, personal history of young-onset breast cancer (≤ 40 years of age), family history of breast cancer, family history of ovarian cancer, and family history of germline mutation were found to be significantly associated with having a pathogenic mutation. (Table 3) The proportion of pathogenic mutations across the Asian ethnicities were consistently more than 25% in this cohort suspected to have hereditary breast cancer syndrome (Fig 2).

**Table 3:** Factors associated with increased odds of harboring a pathogenic mutation (N = 460).

		Univariate Analysis		Multivariate Analysis (Best Model)	
		OR (95% CI)	p-value	OR (95% CI)	p-value
Personal History of	CA Onset ≤40y	1.14 (0.76, 1.69)	0.53		
	Breast CA	0.69 (0.46, 1.03)	0.07		
	**Breast CA Onset ≤40y**	0.81 (0.53, 1.25)	0.35	1.73 (1.01, 2.98)	0.04
	**Ovary CA**	1.42 (0.91, 2.22)	0.13	2.45 (1.40, 2.98)	0.02
	Breast and Ovary CA	2.74 (1.03, 7.25)	0.04		
	**>1 Cancer**	1.52 (0.91, 2.53)	0.11	1.89 (1.08, 3.31)	0.03
Family History of	**FH Breast Yes**	2.10 (1.39, 3.17)	<0.01	2.13 (1.34, 3.37)	<0.01
	**FH Ovary Yes**	4.13 (2.56, 6.67)	<0.01	3.95 (2.32, 6.71)	<0.01
	**FH Germline Mutation**	4.55 (2.43, 9.30)	<0.01	3.56 (1.65, 7.70)	<0.01

#### Variants of uncertain significance

**Fig 3:**
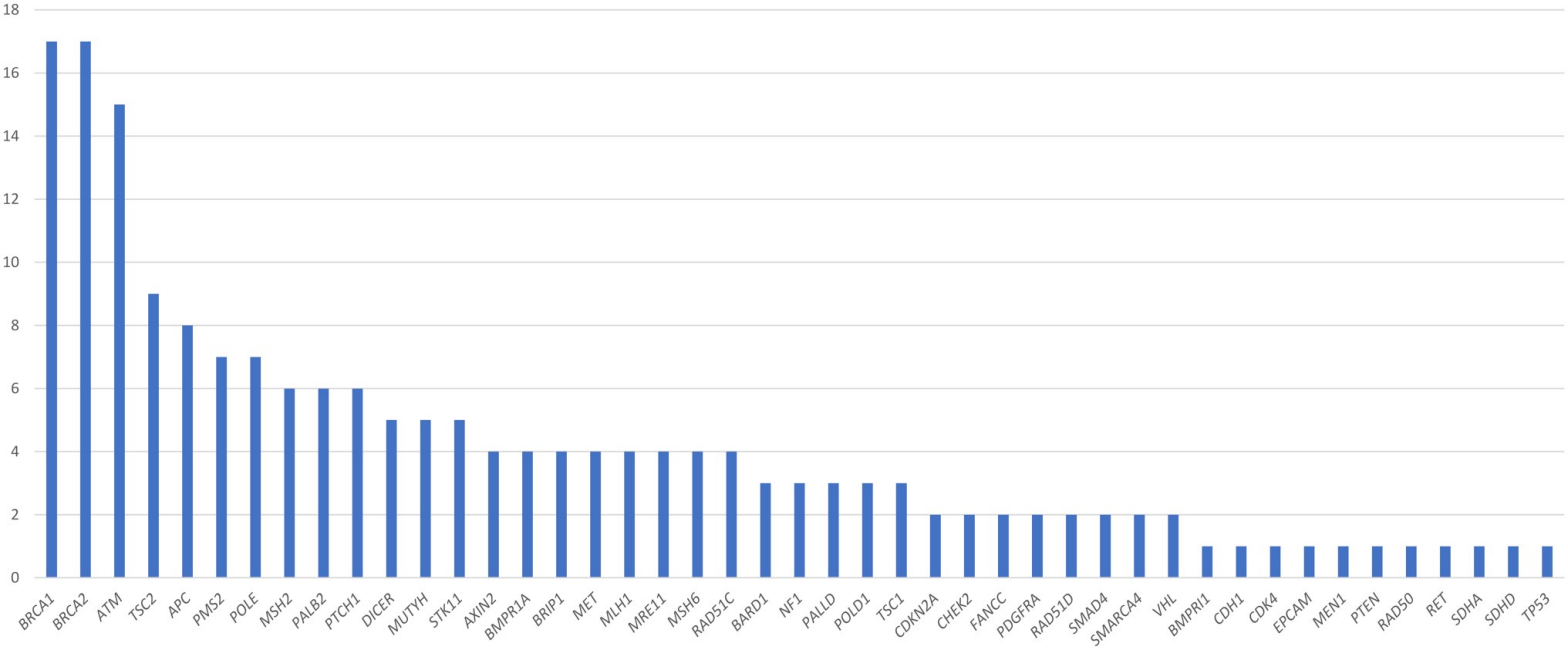
Distribution of variants of uncertain significance (VUS) detected in 125 probands. VUS Count by Gene.

Of 419 probands who underwent targeted or panel testing, 187 VUS across 46 genes were detected in 125 probands on genetic testing (Fig 3). Among probands identified with VUS, 64.8% (81/125) had 1 VUS, 24.8% had 2 VUS, and 10.4% had more than 2 VUS. VUS were most commonly found in *BRCA1* (17/187, 9.1%), *BRCA2* (17/187, 9.1%) and *ATM* (15/187, 8.0%). Testing with a more extensive multigene panel was associated with greater rate of VUS detection, with 44.5% (65/146) of probands tested with a 49-gene panel found to have at least one VUS. There were no statistically significant differences in the frequencies of VUS between patients of different ethnicities.

#### Testing strategy

Excluding the 26 mutation carriers identified via predictive testing, 112 probands were found to have a pathogenic mutation on targeted or panel-based testing. Restricting genetic testing to *BRCA1/2* alone in these 112 probands would identify only 84/112 (75.0%) of mutation carriers. The diagnostic yield is increased to 93/112 (83.0%) if four additional high-penetrance breast cancer genes are included in the test panel (*CDH1*, *PALB2*, *PTEN*, *TP53*), and 100/112 (89.3%) if a broader panel of 15 moderate-penetrance breast-ovarian cancer genes listed in the National Comprehensive Cancer Network guidelines (including *ATM*, *BARD1*, *BRIP1*, *CHEK2*, *NBN*, *NF1*, *RAD51C/51D*, *STK11*) was used (p<0.01). Addition of Lynch Syndrome genes (*MLH1*, *MSH2*, *MSH6*, *PMS2* and *EPCAM*) which are not classical breast cancer predisposition genes but have been shown in several studies to be associated with increased breast cancer risk would identify another 4/112 (3.6%) of mutation carriers (*MLH1* = 3, *MSH2* = 1). [13, 14] Conversely, the VUS detection rate is higher with the number of genes tested: *BRCA1/2* alone (33/419 probands, 7.9%), 6-gene panel (42/419, 10.0%), 15-gene panel (74/419, 17.7%), 20-gene panel including Lynch Syndrome genes (90/419, 21.5%).

## Discussion

This study presents results of clinical germline genetic testing in a large multi-ethnic Asian cohort suspected to have hereditary breast cancer syndrome. Approximately one-third of our 460 probands suspected to have hereditary breast cancer syndrome and who underwent testing, were found to have a pathogenic mutation, similar to reported literature. While *BRCA1/2* remain the most commonly implicated genes in this high-risk cohort, mutations in non-*BRCA1/2* genes were not uncommon (23.2%), and would not have been diagnosed without multigene testing.

Of clinical relevance is the fact that cancer surveillance and risk-reducing guidelines now exist for 13 of the 15 hereditary cancer syndromes caused by non-*BRCA1/2* genes, and would alter management for almost 70% (22/32) of the non-*BRCA1/2* mutation carriers.[8, 15] This is particularly so for high penetrance genes like *TP53* (n = 6) and *MLH/MSH2* (n = 3) which predispose affected individuals to a wide spectrum of malignancies ranging from childhood cancers to colon, adrenal and endocrine cancers in adulthood, and warrant earlier screening strategies and intervention.

However, there is still lack of consensus on the optimal number of candidate genes to be included in panel testing for hereditary breast cancer. Currently, breast cancer-targeted panel testing offered by clinical laboratories typically include up to 9 high and moderate penetrance breast cancer genes (*ATM*, *BRCA1/2*, *CDH1*, *CHEK2*, *NBN*, *PALB2*, *PTEN*, *TP53*) which would identify 85.7% of the mutation carriers seen in our study. Expanding the panel to include 15 breast-ovarian cancer genes and 5 Lynch Syndrome genes listed in the National Comprehensive Cancer Network guidelines for genetic testing in hereditary breast-ovarian cancer would identify 94.6% of mutation carriers in our cohort, and impact on subsequent management. This is significant because Lynch Syndrome genes are not routinely included in multigene panel tests which target hereditary breast cancer alone, and there may not be clinical suspicion of Lynch Syndrome in the absence of colorectal or endometrial cancer. However, multiple studies have indicated that the incidence of breast cancer may be increased in Lynch Syndrome carriers, hence inclusion of the mismatch repair genes may be warranted in probands suspected to have hereditary breast cancer, as seen in our study.[13, 14]

Including more candidate genes may not always be incrementally beneficial. Extensive panel testing covering more than 40 genes is being increasingly marketed, but the diagnostic yield and action-ability remains questionable particularly for candidate genes such as *TSC2 / PALLD* which may not be relevant to patients with suspected hereditary breast cancer. Multigene testing is also associated with increased rates of VUS detection, especially in poorly studied ethnic populations. In our cohort, multigene panel testing was three times more likely to yield VUS in both *BRCA1/2* and non-*BRCA1/2* genes than targeted gene testing. In the clinic setting, VUS are non-informative in risk assessment, and this uncertainty may cause confusion and frustration among patients. Therefore limitation of candidate gene testing would help to reduce potential issues with VUS detection. By restricting testing to 20 cancer predisposition genes including Lynch Syndrome genes which are supported by guidelines, VUS detection could potentially be reduced from 44.5% to 21.5% in our cohort (p<0.01). The additional genes sequenced in more extensive panels such as *RAD50 / FANCC* are at best moderate in penetrance, and would not impact on current management. A guideline-based strategy may be more feasible in detecting clinically actionable mutations, without the drawback of finding VUS in probands.

Incidental findings were also not uncommon with extended panel-based testing in our study. Four individuals with a pathogenic *BRCA1/2* mutation had an unexpected mutation in another gene (two related individuals with *BRCA2* and *RET*, one with *BRCA2* and *MUTYH*, and one with *BRCA1* and *FANCC*) which did not correlate with initial clinical suspicion. An additional six individuals were also found to have pathogenic heterozygous mutations in *MUTYH* in the absence of a personal or family history of colorectal cancer or polyposis. Based on current knowledge, mono-allelic pathogenic mutations in *MUTYH* are unlikely to result in significantly increased risk of cancer, and are thus true incidental findings that do not alter clinical management.[16] However, incidental findings are not necessarily benign in their implications. The accompanying *RET* mutation in the mother-daughter pair with a germline *BRCA2* mutation is highly unusual and cannot be taken lightly. Their family history is positive for breast and ovarian cancer, with no obvious occurrence of medullary thyroid cancer, parathyroid hyperplasia or pheochromocytoma seen in the associated Multiple Endocrine Neoplasia 2 (MEN2) Syndrome.[17] Nevertheless, given that *MEN2* is highly penetrant and affected probands are typically advised to undergo prophylactic thyroidectomy in childhood, appropriate counselling and recommendations to undertake thyroid cancer screening was provided. This is an example of how family history may not always hold key to the underlying genetic predisposition, particularly in the modern era of small nuclear families which make pedigree assessment challenging.[18] This underscores the importance of genetic cancer risk assessment in the pre-test and post-test setting, to complement the additional genetic information provided by multigene testing.

We recognize that the study has limitations due to the heterogeneity of the commercial testing platforms used. Of note, targeted testing of only *BRCA1/2* during the initial phase of the study, and inclusion of related individuals undergoing predictive testing of *BRCA1/2*, may have skewed the proportion of pathogenic *BRCA1/2* mutations detected in our study. Compared to prior reports studying Asian cohorts which reported more frequent *BRCA2* mutations compared to *BRCA1*, the converse was seen in our study population which is more ethnically diverse.[19, 20] This may have been further biased by the inclusion of Caucasian and Middle Eastern patients as our clinic is a regional referral center. If we exclude related individuals, Caucasian and Middle Eastern patients, the proportion of *BRCA1* and *BRCA2* mutations will be 54.9% vs 45.1%, which is comparable to that reported in another smaller study from Singapore.[10]

The disparities between different studies in Asia reflect the real-world scenario faced in Cancer Genetics clinics, where cost and access issues remain significant.[21, 22] To the best of our knowledge, our study presents genetic testing data from the largest Asian cohort of ethnically diverse probands suspected to have hereditary breast cancer syndrome in the clinic, with a significant number undergoing multigene testing comprising a large panel of relevant cancer predisposition genes. While caution should understandably be exercised in using multigene panel testing for suspected hereditary cancer syndromes due to higher rates of uninformative VUS and unwanted incidental findings, its increasing use is inevitable in the real world due to lower test costs and ability to cover differential diagnoses, and this study provides insights on the mutational spectrum in Asian patients suspected with hereditary breast cancer tested with multi-gene panels in the clinic setting.

## Conclusions

We describe the clinical characteristics and mutational profile of patients suspected to have hereditary breast cancer in an Asian academic institution setting. Our study shows that pathogenic mutations could be detected in a third of patients who fulfill clinical guidelines for genetic testing for hereditary breast cancer syndrome, with similar mutation detection rates across diverse Asian ethnic subgroups. Multigene panel tests increased the diagnostic yield, with almost a quarter of the patients diagnosed with pathogenic mutations harboring mutations in non-*BRCA1*/2 genes that could account for their personal/family cancer history, although VUS rate was three times higher than with targeted gene testing. While the number of genes included in multigene panel testing for hereditary breast cancer syndrome is ever increasing, our study showed that limiting the number of genes in the panel to 20 high and moderate penetrance breast-ovarian cancer genes and Lynch Syndrome genes included in established guidelines has the highest yield, while reducing the rates of uninformative VUS to 21.5%. With more widespread use of next generation sequencing technology and unraveling of genetic variants seen in the Asian ethnic population, we expect the understanding of breast cancer predisposition genes in Asia to grow, and management guidelines further tailored to benefit Asian patients.

## Supporting information

S1 Table Cancer predisposition genes tested on multigene panels. (DOC)

S2 Table Clinical characteristics of probands suspected to have hereditary breast cancer syndrome. (DOC)

S3 Table Pathogenic mutations in *BRCA1/2* and non-*BRCA1/2* genes with associated pedigree information. (DOC)
