# Supplementary material for: Discoveries beyond BRCA1/2: Multigene testing in an Asian multi-ethnic cohort suspected of hereditary breast cancer syndrome in the real world
Source: PLoS One. 2019 Mar 15;14(3):e0213746. doi: 10.1371/journal.pone.0213746 (PMC6420039; doi:10.1371/journal.pone.0213746)
Supplement: S1 Table — (DOC) [file pone.0213746.s001.doc]

**S1A Table. High penetrance genes tested on multigene panels**

| High Penetrance Genes | 14-gene Panel | 29-gene Panel | 34-gene Panel | 49-gene Panel |
| --- | --- | --- | --- | --- |
| *BRCA1/2* | ● | ● | ● | ● |
| *CDH1* | ● | ● | ● | ● |
| *PTEN* | ● | ● | ● | ● |
| *TP53* | ● | ● | ● | ● |
| *APC* | ● | ● | ● | ● |
| *MLH1* | ● | ● | ● | ● |
| *MSH2* | ● | ● | ● | ● |
| *MSH6* | ● | ● | ● | ● |
| *PMS2* | ● | ● | ● | ● |
| *MUTYH* | ● | ● | ● | ● |
| *SMAD4* | ● | ● | ● | ● |
| *STK11* | ● | ● | ● | ● |
| *EPCAM* |  | ● | ● | ● |
| *MEN1* |  | ● | ● | ● |
| *MET* |  | ● | ● | ● |
| *RET* |  | ● | ● | ● |
| *VHL* |  | ● | ● | ● |
| *NF1* |  |  | ● | ● |

**S1B Table. Moderate / Low penetrance genes tested on multigene panels**

| Moderate / Low Penetrance Genes | 14-gene Panel | 29-gene Panel | 34-gene Panel | 49-gene Panel |
| --- | --- | --- | --- | --- |
| *ATM* |  | ● | ● | ● |
| *BRIP1* |  | ● | ● | ● |
| *CHEK2* |  | ● | ● | ● |
| *NBN* |  | ● | ● | ● |
| *PALB2* |  | ● | ● | ● |
| *RAD51C* |  | ● | ● | ● |
| *CDK4* |  | ● | ● | ● |
| *CDKN2A* |  | ● | ● | ● |
| *PALLD* |  | ● | ● | ● |
| *PTCH1* |  | ● | ● | ● |
| *BARD1* |  |  | ● | ● |
| *FANCC* |  |  | ● | ● |
| *MRE11A* |  |  | ● | ● |
| *RAD50* |  |  | ● | ● |
| *RAD51D* |  |  |  | ● |
| *AXIN2* |  |  |  | ● |
| *DICER1* |  |  |  | ● |
| *GREM1* |  |  |  | ● |
| *KIT* |  |  |  | ● |
| *PDGFRA* |  |  |  | ● |
| *POLD1* |  |  |  | ● |
| *POLE* |  |  |  | ● |
| *SDHA* |  |  |  | ● |
| *SDHB* |  |  |  | ● |
| *SDHC* |  |  |  | ● |
| *SDHD* |  |  |  | ● |
| *SMARCA4* |  |  |  | ● |
| *TSC1* |  |  |  | ● |
| *TSC2* |  |  |  | ● |
