# Supplementary material for: Discoveries beyond BRCA1/2: Multigene testing in an Asian multi-ethnic cohort suspected of hereditary breast cancer syndrome in the real world
Source: PLoS One. 2019 Mar 15;14(3):e0213746. doi: 10.1371/journal.pone.0213746 (PMC6420039; doi:10.1371/journal.pone.0213746)
Supplement: S2 Table — (DOC) [file pone.0213746.s002.doc]

| **S2 Table. Clinical characteristics of probands suspected to have hereditary breast cancer syndrome. (N=1056)** | | | |
| --- | --- | --- | --- |
| Characteristics |  | No. | (%) |
| Gender (%) | Female | 1002 | (94.9) |
|  | Male | 54 | (5.1) |
|  |  |  |  |
| Age at Time of Visit | Mean (Range) | 47.2y | (12, 91) |
|  | Median | 45y |  |
|  |  |  |  |
| Ethnicity | Chinese | 668 | (63.3) |
|  | Malay | 104 | (9.8) |
|  | Indian | 103 | (9.8) |
|  | Caucasian | 53 | (5.1) |
|  | Indonesian | 47 | (4.5) |
|  | Middle Eastern | 28 | (2.7) |
|  | Filipino | 16 | (1.5) |
|  | Eurasian | 7 | (0.7) |
|  | Myanmese | 7 | (0.7) |
|  | Vietnamese | 6 | (0.6) |
|  | South American | 6 | (0.6) |
|  | Japanese | 3 | (0.3) |
|  | Cambodia | 1 | (0.1) |
|  | Thai | 1 | (0.1) |
|  | Mauritian | 1 | (0.1) |
|  | Not specified | 5 | (0.1) |
|  |  |  |  |
| Number of Cancer Primaries | Cancer-free | 180 | (17.1) |
|  | 1 | 723 | (68.5) |
|  | 2 | 138 | (13.1) |
|  | >2 | 15 | (1.4) |
|  |  |  |  |
| Age of Cancer Onset (n=876) | Mean (Range) | 43.9y | (11, 87) |
|  | Median | 42y |  |
|  |  |  |  |
| Primary Site  (n=876) | Breast | 665 | (75.9) |
|  | Ovary | 156 | (17.8) |
|  | Prostate | 13 | (1.5) |
|  | Colon | 9 | (1.0) |
|  | Endometrium | 8 | (0.9) |
|  | Adrenal | 4 | (0.5) |
|  | Sarcoma | 4 | (0.5) |
|  | Pancreas | 4 | (0.5) |
|  | Thyroid | 3 | (0.3) |
|  | Brain | 2 | (0.2) |
|  | Paraganglioma | 2 | (0.2) |
|  | Cervix | 1 | (0.1) |
|  | Leukaemia | 1 | (0.1) |
|  | Lymphoma | 1 | (0.1) |
|  | Nasopharynx | 1 | (0.1) |
|  | Pituitary | 1 | (0.1) |
|  | Stomach | 1 | (0.1) |
|  |  |  |  |
| Underwent Genetic Testing | Yes | 460 | (43.6) |
|  | No | 596 | (56.4) |
|  |  |  |  |
| Referral Source | Medical Oncology | 671 | (63.5%) |
|  | Surgical Specialties | 162 | (15.3%) |
|  | Self | 65 | (6.2%) |
|  | Gynaecology | 101 | (9.5%) |
|  | General Practitioner | 27 | (2.6%) |
|  | Medical Specialties | 17 | (1.6%) |
|  | Others | 13 | (1.2%) |
