# Supplementary material for: Discoveries beyond BRCA1/2: Multigene testing in an Asian multi-ethnic cohort suspected of hereditary breast cancer syndrome in the real world
Source: PLoS One. 2019 Mar 15;14(3):e0213746. doi: 10.1371/journal.pone.0213746 (PMC6420039; doi:10.1371/journal.pone.0213746)
Supplement: S3 Table — (DOC) [file pone.0213746.s003.doc]

**S3A Table. Pathogenic mutations in *BRCA1* and associated pedigree information (N=66)**

| **ID** | **Gender** | **Race** | **CA Primary** | **Age at diagnosis** | **Gene Affected** | **Nucleotide Change**^†^ | **Amino-acid Change** | **Type of Mutation** | **Family CA History** | **PENN II Score**^‡^ |
| --- | --- | --- | --- | --- | --- | --- | --- | --- | --- | --- |
| 897 | M | Eurasian | CA-free | NA | *BRCA1* | *c.68_69delAG* | *p.E23Vfs*17* | FS Deletion | Sister Bilateral Breast (53), Mother Skin (80’s), Father Lung (85), Pat Uncle Breast (40’s), Pat Cousin Breast (40’s) | 12%  (BRCA: 2%;  BRCA2: 10%) |
| 869 | F | Caucasian | Breast | 37 | *BRCA1* | *c.68_69delAG* | *p.E23Vfs*17* | FS Deletion | Mother Breast (38), Mat Grandmother Ovary (58) | 29%  (BRCA1: 22%;  BRCA2: 7%) |
| 269  (Related to 269) | F | Caucasian | Breast | 38 | *BRCA1* | *c.68_69delAG* | *p.E23Vfs*17* | FS Deletion | Mother Ovary (61), Maternal Aunt Breast (50), Daughter Breast (37) | 29%  (BRCA1: 22%; BRCA2: 7%) |
| 1082 | F | Indian | Breast | 29 | *BRCA1* | *c.68_69delAG* | *p.E23Vfs*17* | FS Deletion | Mother Ovary (42), Mat Grandmother Ovary (40’s) | 46%  (BRCA1: 38%; BRCA2: 8%) |
| 1151 | F | Indian | Ovary | 43 | *BRCA1* | *c.68_69delAG* | *p.E23Vfs*17* | FS Deletion | Sister Ovary (34), Sister Ovary (42), Mat Aunt Ovary (50’s), Mat Aunt Endometrium (75), Father Colon (65), Pat Uncle Throat (40’s), Pat Uncle Kidney (60’s) | NA |
| 766 | F | Chinese | Colon | 53 | *BRCA1* | *c.981_982delAT* | *p.C328** | FS Deletion | Sister Breast (39), Mother Breast (55), Mat Aunt Breast (60’s), Mat Cousin Ovary (55). May Cousin Breast (28), Mat Cousin Lung (60’s), Pat Aunt Breast, Pat Cousin Breast, | 21%  (BRCA1: 17%; BRCA2: 4%) |
| 419 | F | Chinese | Breast | 31 | *BRCA1* | *c.1847delC* | *p.S616Lfs* | FS Deletion | Mother Ovary (64), Mat Aunt Endometrium (40’s), Mat Cousin Breast (35), Mat Grandmother Ovary (40’s) | 47%  (BRCA1: 39%; BRCA2: 8%) |
| 1115 | F | Caucasian | Breast | 41 | *BRCA1* | *c.2071delA* | *p.R691Dfs*10* | FS Deletion | Sister Ovary (45), Father Colon (63), Pat Grandmother Ovary (52), Pat Grandaunt Gastric, Mat Aunt Liver (50), Mat Aunt Breast (65), Mat Uncle Prostate (70), Mat Grandmother Intestine (50’s) | 18%  (BRCA1: 10%; BRCA2: 8%) |
| 98 | F | Chinese | Breast | 38 | *BRCA1* | *c.2253_2254delGT* | *p.M751Ifs* | FS Deletion | Mat Aunt Breast (60), Father Lung, Pat Cousin Breast (48), Pat Cousin Brain (60), Pat Cousin Colon (60), Pat Cousin Colon (73) | 23%  (BRCA1: 17%; BRCA2: 6%) |
| 712 | F | Chinese | Breast | 58 | *BRCA1* | *c.2397delA* | *p.G800Vfs* | FS Deletion | Sister Bilateral Breast (39, 54) / Sarcoma (50) | 22%  (BRCA1:14%; BRCA2: 8%) |
| 713  (Related to 712) | F | Chinese | Breast | 39 | *BRCA1* | *c.2397delA* | *p.G800Vfs* | FS Deletion | Sister Breast / Ovary (58) | 22%  (BRCA1: 14%; BRCA2: 8%) |
| 735 | F | Chinese | Ovary | 39 | *BRCA1* | *c.2866_2870delTCTCA* | *p.S956Vfs*13* | FS Deletion | Father Prostate (60’s), Pat Aunt Ovary (40’s), Pat Grandmother NPC (40’s), Pat Grandfather Prostate (80’s), Mat Uncle Lung (60), Mat Cousin Breast (30’s) | 21%  (BRCA1: 14%;  BRCA2: 7%) |
| 1341 | F | Chinese | Breast | 39 | *BRCA1* | *c.3214delC* | *p.L1072fs* | FS Deletion | Pat Aunt Breast (42) | 12%  (BRCA1: 6%;  BRCA2: 6%) |
| 440 | F | Chinese | Breast | 44 | *BRCA1* | *c.3214delC* | *p.L1072fs* | FS Deletion | Sister Breast (40’s), Sister Breast (40’s) | 20%  (BRCA1: 15%; BRCA2: 5%) |
| 14 | F | Middle Eastern | Breast | 35 | *BRCA1* | *c.3756_3759delGTCT* | *p.S1253Rfs* | FS Deletion | No FH | 13%  (BRCA1: 6%;  BRCA2: 7%) |
| 925 | F | Caucasian | Ovary | 48 | *BRCA1* | *c.3916_3917delTT* | *p.L1306Dfs*23* | FS Deletion | Mother Ovary (53), Sister Breast (27), Mat Uncle Throat (60’s), Mat Uncle Throat (50’s), Pat Uncle Gastric (50’s), Pat Uncle Gastric (50’s), Pat Uncle Lung (50’s) | 49%  (BRCA1: 40%; BRCA2: 9%) |
| 487 | F | Chinese | CA-free | NA | *BRCA1* | *c.4065_4068delTCAA* | *p.N1355Kfs* | FS Deletion | Mother Ovary (44) / Colon (58), Sister Ovary (39) / Bladder (41), Sister Ovary (40) | NA |
| 1500 | F | Indian | Breast | 54 | *BRCA1* | *c.4065_4068delTCAA* | *p.N1355Kfs* | FS Deletion | Sister Breast ( 40), Niece Breast (31), Mother Cervix (50’s) | 17%  (BRCA1: 9%;  BRCA2: 8%) |
| 28 | F | Middle Eastern | Breast | 31 | *BRCA1* | *c.4065_4068delTCAA* | *p.N1355Kfs* | FS Deletion | Mother Breast (41), Pat Uncle Prostate (80) | 22%  (BRCA1: 14%; BRCA2: 8%) |
| 329  (Related to 28) | F | Middle Eastern | Breast | 39 | *BRCA1* | *c.4065_4068delTCAA* | *p.N1355Kfs* | FS Deletion | Daughter Breast (31), Pat Aunt Breast (60’s), Pat Cousin Abdomen (60’s) | 24%  (BRCA1: 14%; BRCA2: 10%) |
| 1215  (Related to 28) | F | Middle Eastern | CA-free | NA | *BRCA1* | *c.4065_4068delTCAA* | *p.N1355Kfs* | FS Deletion | Sister Breast (31), Mother Breast (41), Pat Uncle Prostate (80) | 11%  (BRCA1: 7%;  BRCA2: 4%) |
| 442 | F | Indonesian | Breast | 55 | *BRCA1* | *c.4386del* | *p.E1462Dfs*4* | FS Deletion | Sister Ovary (46), Sister Ovary (54), Pat Uncle Prostate (70’s). Mat Cousin Colon (40) | 29%  (BRCA1: 25%; BRCA2: 4%) |
| 1099 | F | Caucasian | CA-free | NA | *BRCA1* | *c.5030_5033delCTAA* | *p.T1677Ifs* | FS Deletion | Pat Uncle Prostate (58), Pat Grandmother Ovary (87), Pat Granduncle Prostate (76), Pat Granduncle Prostate (74) / Liver (81). Pat Grandaunt Thyroid (47), Pat Aunt Breast (52), Pat Great Grandmother Breast (50) / Biliary (91), Pat Great Grandfather Colon (69) | 17%  (BRCA1: 6%;  BRCA2: 11%) |
| 258 | F | Caucasian | Breast | 35 | *BRCA1* | *c.5137delG* | *p.V1713** | FS Deletion | Father Throat (55), Mat Grandmother Unknown | 13%  (BRCA1: 6%;  BRCA2: 7%) |
| **926** | **F** | **Chinese** | **Ovary** | **64** | ***BRCA1***  ***FANCC*** | ***c.5525delT***  ***c.1290C>A*** | ***p.V842Efs*131***  ***p.Y430**** | **FS Deletion**  **Nonsense** | **Sister Oral Cavity (42), Mother Colon / Endometrium (55)** | **NA** |
| **90** | **F** | **Caucasian** | **Breast** | **30** | ***BRCA1*** | ***c.4416_4417delinsG*** | ***p.S1473Lfs*32*** | **FS Deletion / Insertion** | **Mother Ovary (31)** | **26%**  **(BRCA1: 18%; BRCA2: 8%)** |
| **1180** | **F** | **Indian** | **Breast** | **35** | ***BRCA1*** | ***c.5365_5366delinsA*** | ***A1789Ifs*4*** | **FS Deletion / Insertion** | **Father Colon (50’s), Mother Breast (30’s)** | **18%**  **(BRCA1: 11%; BRCA2: 7%)** |
| 934 | F | Indonesian | Breast | 37 | *BRCA1* | *c.329insA* | *p.E111Gfs*3* | FS insertion | Mother Breast (42), Mat Aunt Ovary (77), Mat Aunt Breast (45), Mat Aunt Breast (42), Mat Aunt Breast, Mat Cousin (40), Mat Cousin Breast (50), Mat Cousin Breast (50), Mat Bilateral Breast (48, 60), Mat Cousin Breast (48), Mat Cousin Ovary (29) | 82%  (BRCA1: 74%; BRCA2: 8%) |
| 1141 | F | Malay | Breast | 41 | *BRCA1* | *c.2726insA* | *p.N909Kfs*6* | FS insertion | Sister Ovary (43), Sister Ovary (43), Mother Colon (60’s), Mat Aunt Cervix (40’s) | 28%  (BRCA1: 21%; BRCA2: 7%) |
| 1157  (Related to 1141) | F | Malay | Ovary | 43 | *BRCA1* | *c.2726insA* | *p.N909Kfs*6* | FS insertion | Sister Breast (41), Sister Ovary (43), Mother Colon (60’s), Mat Aunt Cervix (40’s) | 28%  (BRCA1: 21%; BRCA2: 7%) |
| 1152 | F | Malay | Ovary | 44 | *BRCA1* | *c.2726insA* | *p.N909Kfs*6* | FS insertion | No FH | 14%  (BRCA1: 9%;  BRCA2: 5%) |
| 97 | F | Indonesian | Ovary | 58 | *BRCA1* | *c.2726insA* | *p.N909Kfs*6* | FS insertion | Mother Breast (28), Mat Aunt Breast (55), Mat Aunt Breast (60), Mat Cousin Breast (35), Nephew Seminoma (21) | 31%  (BRCA1: 23%, BRCA2: 8%) |
| 304 | F | Eurasian | CA-free | NA | *BRCA1* | Duplication of exon 12 (3 copies) | Duplication | Duplication | Mother Ovary (57), Mat Grandaunt Ovary (60’s), Mat Grandaunt Breast (30’s), Mat Great Grandmother Unknown | 19%  (BRCA1: 15%; BRCA2: 4%) |
| 528  (Related to 304) | F | Caucasian | Ovary | 57 | *BRCA1* | Duplication of Exon 12 (3 copies) | Duplication | Duplication | Mat Aunt Ovary (60’s), Mat Aunt Breast (30’s). Mat Grandmother Unknown | 21%  (BRCA1: 14%; BRCA2: 7%) |
| 1105 | F | Indian | Breast | 43 | *BRCA1* | *c.631_632insT* | *p.G211Vfs*5* | FS Insertion | Sister Ovary (40), Father Throat (63), Nephew Leukaemia (3) | 15%  (BRCA1: 9%;  BRCA2: 6%) |
| 428 | F | Malay | Breast | 42 | *BRCA1* | *c.2845_2846insA* | *p.N909Kfs* | FS Insertion/ | Mother Breast (60's) | 13%  (BRCA1: 7%;  BRCA2: 6%) |
| 17 | F | Malay | Breast | 38 | *BRCA1* | *c.2845_2846insA* | *p.N909Kfs* | FS Insertion | Mother Ovary (48), Mat Grandaunt Ovary (50) | 32%  (BRCA1: 26%;  BRCA: 6%) |
| 313 | F | Chinese | Ovary | 42 | *BRCA1* | *c.213-12A>G* | *p.R71Sfs*21* | Intronic change | Father Esophagus (76), Pat Cousin Lymphoma (50’s) | NA |
| 1193 | F | Chinese | Breast | 35 | *BRCA1* | *c.213-12A>G* | *p.R71Sfs*21* | Intronic Change | Sister Breast (43), Mother Bilateral Breast (50’s / 60’s), Mat Aunt Breast | 22%  (BRCA1: 11%; BRCA2: 11%) |
| 1287 | F | Chinese | Ovary | 49 | *BRCA1* | Deletion of Exon 3 | Deletion | Large deletion | Sister Breast (46), Sister Breast (44), Pat uncle Skin (60), Mat Aunt Ovary, Mat Aunt Endometrium (40’s), Mat Cousin Endometrium, Mat Cousin Prostate, Mat Cousin Colon, Mat Cousin Colon | 14%  (BRCA1: 9%;  BRCA2: 5%) |
| 806  (Related to 1287) | F | Chinese | Breast | 46 | *BRCA1* | Deletion of Exon 3 | Deletion | Large deletion | Sister Ovary (49), Sister Breast (44), Pat Uncle Skin (60), Mat Aunt Ovary, Mat Aunt Endometrium (40’s), Mat Cousin Prostate, Mat Cousin Colon | 30%  (BRCA1: 23%; BRCA2: 7%) |
| **433** | **F** | **Indian** | **CA-free** | **NA** | ***BRCA1*** | **Deletion of Exons 13-15** | **Deletion** | **Large deletion** | **Mother Ovary (55), Mat Aunt Breast (60), Mat Aunt Breast (66), Mat Uncle Colon (64), Mat Cousin Breast (35), Mat Cousin Ovary (40), Mat Cousin Breast, Mat Cousin Ovary, Mat Grandfather Leukaemia (70)** | **36%**  **(BRCA1: 32%; BRCA2: 4%)** |
| **525**  **(Related to 433)** | **F** | **Indian** | **CA-free** | **NA** | ***BRCA1*** | **Deletion of Exons 13-15** | **Deletion** | **Large deletion** | **Mat Aunt Breast (35), Mat Aunt Breast, Mat Aunt Ovary (40), Mat Aunt Ovary, Mat Grandmother Ovary (55), Mat Grandaunt Breast (60), Mat Granduncle Colon (64)** | **4%**  **(BRCA1: 2%;**  **BRCA2: 2%)** |
| **1200**  **(Related to 433)** | **F** | **Indian** | **Ovary** | **55** | ***BRCA1*** | **Deletion of Exons 13-15** | **Deletion** | **Large deletion** | **Sister Breast (60), Sister Breast (66), Brother Colon (64), Father Leukaemia (70), Niece Breast (35), Niece Ovary (40), Niece Ovary (40)** | **59%**  **(BRCA1: 52%; BRCA2: 7%)** |
| **896** | **F** | **Indonesian** | **Ovary** | **41** | ***BRCA1*** | **Deletion of Exons 13-15** | **Deletion** | **Large deletion** | **Mat Uncle Lung (59), Pat Cousin Blood (41), Pat Cousin Blood (47)** | **NA** |
| 1413 | F | Chinese | Ovary | 50 | *BRCA1* | *c.5072C>A* | *p.T1691K* | Missense | Mat Cousin Breast (50’s), Father Throat (50’s) | 8%  (BRCA1: 4%;  BRCA2: 4%) |
| 1154 | F | Malay | Ovary | 47 | *BRCA1* | *c.5165C>T* | *p.S1722F* | Missense | Sister Breast (40), Brother Lung (40’s), Father Lung (60’s) | 17%  (BRCA1: 11%; BRCA2: 6%) |
| 1280 | F | Indian | Ovary | 35 | *BRCA1* | *c.5074+1G>A* | *Splice Site Mutation* | Splice Site Mutation | Mat Cousin Unknown, Mat Cousin Unknown (50’s), Mat Grandmother Ovary (60’s) | NA |
| 1066 | F | Caucasian | Cervix | 33 | *BRCA1* | *c.427G>T* | *p.E143** | Nonsense | Pat Aunt Lung (40’s), Pat Grandfather Lung (60’s) / Prostate (69), Pat Uncle Prostate (59), Mat Grandaunt Breast (46), Mat Great Grandfather Colon (40’s) | 2%  (BRCA1: 1%;  BRCA2: 1%) |
| 1064 | F | Chinese | CA-free | NA | *BRCA1* | *c.2635G>T* | *p.E879** | Nonsense | Mother Bilateral Breast (30’s, 60), Mat Aunt Breast (46), Mat Cousin Breast (29), Mat Uncle Colon (63), Mat Grandfather Liver (49) | 13%  (BRCA1: 9%;  BRCA2: 4%) |
| 634  (Related to 1064) | F | Chinese | Breast | 35 | *BRCA1* | *c.2635G>T* | *p.E879** | Nonsense | Sister Breast (46), Father Liver (49) | 14%  (BRCA1: 7%;  BRCA2: 7%) |
| 636  (Related to 1064) | F | Chinese | Breast | 46 | *BRCA1* | *c.2635G>T* | *p.E879** | Nonsense | Sister Breast (35), Father Liver (49) | 14%  (BRCA1: 7%;  BRCA2: 7%) |
| 635  (Related to 1064) | F | Chinese | CA-free | NA | *BRCA1* | *c.2635G>T* | *p.E879** | Nonsense | Sister Breast (46), Sister Breast (35), Father Liver (49) | 8%  (BRCA1: 4%;  BRCA2: 4%) |
| 473  (Related to 1064) | F | Chinese | Breast | 29 | *BRCA1* | *c.2635G>T* | *p.E879** | Nonsense | Mother Breast (46), Mat Aunt Bilateral Breast (33, 58), Mat Grandfather Liver (49) | 26%  (BRCA1: 18%; BRCA2: 8%) |
| 1607 | F | Indonesian | Breast | 44 | *BRCA1* | *c.3607C>T* | *p.R1203** | Nonsense | Mother Ovary (47), Mat Aunt Breast (60), Mat Aunt Breast (32), Mat Grandmother Cervix (30’s) | 27%  (BRCA1: 19%; BRCA2: 8%) |
| 1440 | F | Chinese | CA-free | NA | *BRCA1* | *c.3661G>T* | *p.E1221** | Nonsense | Sister Breast (40’s) / Brain (50’s), Mother Cervix (40), Mat Cousin Breast (26), Mat Cousin Breast, Mat Cousin Breast, Mat Cousin Breast | 21%  (BRCA1: 12%; BRCA2: 9%) |
| 1586 | F | Chinese | Ovary | 54 | *BRCA1* | *c.3661G>T* | *p.E1221** | Nonsense | Sister Ovary (30’s), Sister Breast (30’s), Mother Pancreas (71), Mat Cousin NPC, Pat Uncle Prostate | 47%  (BRCA1: 30%; BRCA2: 17%) |
| 1358 | F | Chinese | Ovary | 39 | *BRCA1* | *c.4201C>T* | *p.Q1401** | Nonsense | Sister Ovary (57), Mother Colon (54), Mat Aunt Ovary (62), Mat Aunt Ovary (66), Mat Aunt Breast (50), Mat Aunt Breast (48), Mat Uncle Prostate (70’s), Mat Cousin Bilateral Breast (30’s), Mat Cousin Breast (42), Mat Cousin Ovary (38), Mat Cousin Breast (51), Mat Cousin Endometrium (50’s), Mat Grandmother Kidney, Mat Grandaunt Ovary / Endometrium, Mat Grandaunt Ovary | 124%  (BRCA1: 98%; BRCA2: 26%) |
| 1359  (Related to 1358) | F | Chinese | Ovary | 57 | *BRCA1* | *c.4201C>T* | *p.Q1401** | Nonsense | Sister Ovary (39), Mother Colon (54), Mat Aunt Ovary (62), Mat Aunt Ovary (66), Mat Aunt Breast (50), Mat Aunt Breast (48), Mat Uncle Prostate (70’s), Mat Cousin Bilateral Breast (30’s), Mat Cousin Breast (42), Mat Cousin Ovary (38), Mat Cousin Breast (51), Mat Cousin Endometrium (50’s), Mat Grandmother Kidney, Mat Grandaunt Ovary / Endometrium, Mat Grandaunt Ovary | 124%  (BRCA1: 98%; BRCA2: 26%) |
| 953 | F | Chinese | Breast | 33 | *BRCA1* | *c.4327C>T* | *p.R1443** | Nonsense | Sister Breast (34), Mother Breast (51) / Peritoneum (76), Mat Aunt Bilateral Breast (41, 46) / Pancreas (67), Mat Cousin Ovary (44), Mat Grandmother Ovary (58), Mat Cousin Colon (70’s), Mat Cousin Breast, Father Esophagus | 118%  (BRCA1: 92%; BRCA2: 26%) |
| 252  (Related to 953) | F | Chinese | CA-free | NA | *BRCA1* | *c.4327C>T* | *p.R1443** | Nonsense | Mother Bilateral Breast (33, 42) / Peritoneum (51), Mat Aunt Breast (34), Mat Grandmother Breast (51), Mat Grandaunt Bilateral Breast (41, 46) / Pancreas (67), Mat Aunt Ovary (44), Mat Great Grandmother Ovary (58), Mat Great Granduncle Colon (70’s), Mat Grandaunt Colon (70’s), Mat Aunt Breast, Mat Grandfather Esophagus | 47%  (BRCA1: 42%; BRCA2: 5%) |
| 1639  (Related to 953) | F | Chinese | CA-free | NA | *BRCA1* | *c.4327C>T* | *p.R1443** | Nonsense | Mother Breast (35), Mat Aunt Bilateral Breast (33, 42) / Peritoneum (49), Mat Grandmother Bilateral Breast (50, 60), Mat Grandaunt Bilateral Breast (41, 46) / Pancreas (67), Mat Aunt Ovary (44) | 43%  (BRCA1: 32%; BRCA2: 11%) |
| 951  (Related to 953) | M | Chinese | CA-free | NA | *BRCA1* | *c.4327C>T* | *p.R1443** | Nonsense | Sister Breast (34), Sister Bilateral Breast (33, 42) / Peritoneum (51), Mother Breast (51) / Peritoneum (76), Mat Aunt Bilateral Breast (41, 46) / Pancreas (67), Mat Cousin Ovary (44), Mat Grandmother Ovary (58), Mat Aunt Colon (70’s), Mat Aunt Breast, Father Esophagus | 59%  (BRCA1: 46%; BRCA2: 13%) |
| 366 | F | Chinese | Breast | 37 | *BRCA1* | *c.4801A>T* | *p.K1601** | Nonsense | Mother Breast (43) | 17%  (BRCA1: 10%; BRCA2: 7%) |
| 518 | F | Chinese | Breast | 49 | *BRCA1* | *c.5353C>T* | *p.Q1785** | Nonsense | Sister Breast (37), Mother Breast (44) / Ovary, Mat Grandmother Ovary (60’s) | 37%  (BRCA1: 30%; BRCA2: 7%) |
| 80 | F | Mauritian | Breast | 34 | *BRCA1* | *c.5503C>T* | *p.R1835** | Nonsense | Pat Cousin Breast (34), Pat Grandmother Breast (30’s) | 16%  (BRCA1: 9%;  BRCA2: 7%) |

^†^ Novel mutations are underlined and in bold.

^‡^ The PENN II Score predicts the pre-test probability (prior probability) that a person has a BRCA1 or BRCA2 mutation. University of Pennsylvania Abramson Cancer Center, The Penn II BRCA1 and BRCA2 Mutation Risk Evaluation Model. Philadelphia, PA https://www.afcri.upenn.edu:8022/itacc/penn2/index.asp .

Abbreviations: AML, acute myeloid leukemia; CA, cancer; FH, family history; FS, frameshift; HCC, hepatocellular carcinoma; Mat, maternal; NPC, nasopharyngeal carcinoma; Pat, paternal; RCC, renal cell carcinoma

**S3B Table. Pathogenic mutations in *BRCA2* and associated pedigree information (N=44)**

| **ID** | **Gender** | **Race** | **CA Primary** | **Age at diagnosis** | **Gene Affected** | **Nucleotide Change**^†^ | **Amino-acid Change** | **Type of Mutation** | **Family CA History** | **PENN II Score** |
| --- | --- | --- | --- | --- | --- | --- | --- | --- | --- | --- |
| 529 | F | Indonesian | Ovary | 48 | *BRCA2* | *c.262_263delCT* | *p.L88Afs*12* | FS Deletion | Sister Breast (45), Mother Endometrium (38), Mat Aunt Liver (42), Mat Aunt Breast (60), Mat Grandmother Unknown, Pat Uncle Lung (72), Pat Uncle Liver (62), Pat Aunt Ovary (63) | 13%  (BRCA1: 8%;  BRCA2: 5%) |
| 59 | F | Chinese | Breast | 37 | *BRCA2*  *MUTYH* | *c.774_775delAA*  *c.934-2A>G* | *p.Q260Sfs*15*  *Splice acceptor in Intron 10* | FS Deletion  Splice Site Error | Mat Aunt Breast (50s), Pat Grandmother Throat (70s), Pat Cousin Breast, Pat Cousin Breast | 15%  (BRCA1: 8%;  BRCA2: 7%) |
| 317 | F | Indonesian | Breast | 35 | *BRCA2* | *c.771_775delTCAAA* | *p.N257Kfs*17* | FS Deletion | Sister Breast (53), Sister Breast (37). Mother Breast (48), Father Lung (59) | 11%  (BRCA1: 7%;  BRCA2: 4%) |
| 1503 | F | Chinese | CA-free | NA | *BRCA2* | *c.862_868delCCAAATG* | *p.P288Sfs* | FS Deletion | Sister Bilateral Breast (35, 44), Sister Ovary (40), Mother Breast (38) | 18%  (BRCA1: 13%; BRCA2: 5%) |
| 1508  (Related to 1503) | F | Chinese | CA-free | NA | *BRCA2* | *c.862_868delCCAAATG* | *p.P288Sfs* | FS Deletion | Sister Bilateral Breast (35, 44), Sister Ovary (40), Mother Breast (38) | 18%  (BRCA1: 13%; BRCA2: 5%) |
| 1507  (Related to 1503) | F | Chinese | Breast | 35 | *BRCA2* | *c.862_868delCCAAATG* | *p.P288Sfs* | FS Deletion | Sister Ovary (40), Mother Breast (38) | 34%  (BRCA1: 25%; BRCA2: 9%) |
| 974 | F | Chinese | Breast | 53 | *BRCA2* | *c.1763_1766delATAA* | *p.N588Sfs*25* | FS Deletion | Mother Breast (41), Brother HCC (46) | 13%  (BRCA1: 7%;  BRCA2: 6%) |
| 1195 | F | Chinese | Breast | 36 | *BRCA2* | *c.1773_1776delTTAT* | *p.I591Mfs* | FS Deletion | No FH | 13%  (BRCA1: 6%;  BRCA2: 7%) |
| **422** | **F** | **Chinese** | **Breast** | **45** | ***BRCA2*** | ***c.2095_2096delCA*** | ***p.E699Vfs*** | **FS Deletion** | **Mat Uncle Liver** | **27%**  **(BRCA1: 22%; BRCA2: 5%)** |
| 173 | F | Chinese | Breast | 32 | *BRCA2* | *c.2442delC* | *p.M815Wfs* | FS Deletion | No family history | 15%  (BRCA1: 7%;  BRCA2: 8%) |
| 1049 | F | Eurasian | Ovary | 67 | *BRCA2* | *c.2442delC* | *p.M815Wfs* | FS Deletion | No FH | NA |
| 1002 | F | Malay | CA-free | NA | *BRCA2* | *c.2808_2811delACAA* | *p.A938Pfs* | FS Deletion | Mother Bilateral Breast (40, 44), Mat Grandmother Peritoneal (60), Mat Grandaunt Bilateral Breast (30’s), Mat Cousin Breast (41), Pat Uncle Oral Cavity (50’s), Pat Aunt Breast (60’s) | 11%  (BRCA1: 5%;  BRCA2: 6%) |
| 999  (Related to 1002) | F | Malay | Breast | 40 | *BRCA2* | *c.2808_2811delACAA* | *p.A938Pfs* | FS Deletion | Mat Aunt Bilateral Breast (30’s), Mat Cousin Breast (41), Mother Peritoneum | 20%  (BRCA1: 9%;  BRCA2: 11%) |
| 1574 | F | Chinese | Breast | 33 | *BRCA2* | *c.2808_2811delACAA* | *p.A938Pfs* | FS Deletion | Mat Cousin Breast (37), Pat Grandmother Breast (50), Pat Aunt Breast | 18%  (BRCA1: 11%; BRCA2: 7%) |
| 68 | F | Indian | Ovary | 72 | *BRCA2* | *c.3865_3868delAAAT* | *p.K1289Afs* | FS Deletion | Half-sister Gastric, Son Breast (50’s) | 16%  (BRCA1: 4%;  BRCA2: 12%) |
| 411 | F | Middle Eastern | Ovary | 62 | *BRCA2* | ***c.4124delA*** | ***p.E1375Gfs*** | FS Deletion | Daughter Breast (30), Pat Aunt Breast | 26%  (BRCA1: 18%; BRCA2: 8%) |
| 353 | F | Chinese | Ovary | 43 | *BRCA2* | *c.5351delA* | *p.N1784Tfs* | FS Deletion | Sister Ovary (38), Mother Gastric (67), Mat Uncle Liver, Mat Uncle Liver, Mat Uncle Liver, Mat Cousin Unknown, Mat Grandfather Liver, Pat Grandfather Unknown | NA |
| 563 | M | Chinese | Prostate | 66 | *BRCA2* | *c.5642_5645delAATC* | *p.S1882Kfs* | FS Deletion | Brother Lung (70). Sister Sarcoma (48), Sister Lung (65), Brother Pancreas (75), Niece Breast (44) | 22%  (BRCA1: 4%;  BRCA2: 18%) |
| 566  (Related to 563) | F | Chinese | Breast | 44 | *BRCA2* | *c.5642_5645delAATC* | *p.S1882Kfs* | FS Deletion | Father Pancreas (75), Pat Aunt Sarcoma (48), Pat Aunt Lung (60’s), Pat Uncle Lung (70), Par Uncle Prostate (66) | 22%  (BRCA1: 4%;  BRCA2: 18%) |
| 509 | M | Caucasian (Ashkenazi) | CA-free | NA | *BRCA2* | *c.6174delT* | *p.F2058Lfs* | FS Deletion | Mother Breast (43) | 14%  (BRCA: 9%;  BRCA2: 5%) |
| 338 | F | Caucasian | CA-free | NA | *BRCA2* | *c.6275_6276delTT* | *p.L2092Pfs* | FS Deletion | Mother Breast (38), Mat Grandmother Breast (36), Mat Grandaunt Breast (44), Mat Grandaunt Breast (49), Mat Grandaunt Breast (65) | 12%  (BRCA1: 8%;  BRCA2: 4%) |
| 612 | F | Caucasian | Breast | 41 | *BRCA2* | *c.6275_6276delTT* | *p.L2092Pfs* | FS Deletion | Father Prostate (67), Pat Uncle Lung (60), Pat Grandmother Ovary, Mat Aunt Breast (60’s) | 18%  (BRCA1: 10%; BRCA2: 8%) |
| 589 | F | Chinese | Breast | 57 | *BRCA2* | *c.6405_6409delCTTAA* | *p.N2135Kfs*3* | FS Deletion | Sister Breast (40) / Lung (61), Father Colon (51), Pat Grandmother Unknown | 11%  (BRCA1: 5%;  BRCA2: 6%) |
| **38** | **F** | **South American** | **CA-free** | **NA** | ***BRCA2*** | ***c.7819delA*** | ***p.T2607Lfs*** | **FS Deletion** | **Father Unkown (70’s), Pat Uncle Unknown (60’s), Pat Aunt Breast (30’s), Pat Aunt Breast (40’s), Pat Aunt Breast (40’s), Pat Aunt Breast (40’s), Mat Aunt Liver (40’s), Mat Grandmother Liver (80’s)** | **17%**  **(BRCA1: 10%;**  **BRCA2: 7%)** |
| 1366 | F | Chinese | Breast | 44 | *BRCA2* | *c.8585_8586delTA* | *p.L2862Rfs*6* | FS Deletion | Sister Colon (38), Mother Ovary (50’s), Father Lung (60’s), Mat Aunt Breast (51) | 14%  (BRCA1: 9%;  BRCA2: 5%) |
| 859 | F | Chinese | Breast | 40 | *BRCA2* | *c.8915delT* | *p.L2972Cfs* | FS Deletion | Sister Ovary | 36%  (BRCA1: 28%; BRCA2: 8%) |
| 123 | F | Chinese | Breast | 44 | *BRCA2* | *c.8961_8964delGAGT* | *p.S2988Ffs* | FS Deletion | Brother NPC (40’s), Mother Breast (54) / Tongue (63) | 13%  (BRCA1: 6%;  BRCA2: 7%) |
| 1541 | F | Chinese | Breast | 25 | *BRCA2* | *c.9414_9415delAT* | *p.L3138fs* | FS Deletion | Mother Breast (30), Mat Aunt Breast (40) | 31%  (BRCA1: 22%; BRCA2: 9%) |
| 319 | M | Indonesian | Breast | 54 | *BRCA2* | *c.2471_2476delTAAATG* | *p.L824** | Non-FS Deletion | Pat Grandmother Breast (60) | 14%  (BRCA1: 2%;  BRCA2: 12%) |
| 1209 | M | Chinese | Breast | 64 | *BRCA2* | *c.581G>A* | *p.W194** | Nonsense | Sister Throat (40) | 10%  (BRCA1: 1%;  BRCA2: 9%) |
| 281 | F | Chinese | Ovary | 46 | *BRCA2* | *c.3109C>T* | *p.Q1037** | Nonsense | Mother Bilateral Breast (56, 60) / Lymphoma (66), Mat Aunt NPC (50’s), Mat Aunt Unknown, Mat Uncle NPC (50’s), Father Colon (76), Pat Uncle Pancreas (60’s) | 11%  (BRCA1: 5%;  BRCA2: 6%) |
| 809 | F | Chinese | Breast | 39 | *BRCA2* | *c.3109C>T* | *p.Q1037** | Nonsense | Mother Bilateral Breast (57, 65), Mat Aunt Bilateral Breast (50’s), Mat Grandmother Breast (50’s) | 18%  (BRCA1: 8%;  BRCA2: 10%) |
| 573 | F | Chinese | Breast | 45 | *BRCA2* | *c.3109C>T* | *p.Q1037** | Nonsense | Pat Aunt Breast (50’s), Pat Grandmother Throat | 8%  (BRCA1: 3%;  BRCA2: 5%) |
| 40 | F | Chinese | CA-free | NA | *BRCA2*  *RET*^‡^ | *c.5645C>A*  *c.2410G>A*^‡^ | *p.S1882**  *p.V804M*^‡^ | Nonsense  Missense | Mother Breast (49), Mat Grandmother Bone Marrow (47), Mat Grandaunt Breast (50s), Mat Grandaunt Ovary (40s), Mat Granduncle Liver (60s), Mat Great Grandmother Unknowm, Pat Aunt Hodgkin’s (23) / Thyroid (39), Pat Grandfather Gastric (69), Pat Grandmother Pancreas (50) | 12%  (BRCA1: 7%;  BRCA2: 5%) |
| 648  (Related to 40) | F | Asian (Others) | Breast | 49 | *BRCA2*  *RET*^‡^ | *c.5645C>A*  *c.2410G>A*^‡^ | *p.S1882**  *p.V804M*^‡^ | Nonsense  Missense | Mother Breast (47), Mat Aunt Breast (50s), Mat Aunt Ovary (40s), Mat Uncle Liver (60s), Mat Grandmother Unknown | 12%  (BRCA1: 7%;  BRCA2: 5%) |
| 41 | F | Chinese | Ovary | 41 | *BRCA2* | *c.5656C>T* | *p.Q1886** | Nonsense | Sister Breast (42), Mat Cousin Leukaemia (20’s), Mat Cousin Breast (40), Pat Grandfather Breast | 19%  (BRCA1: 13%; BRCA2: 6%) |
| 1496 | F | Filipino | Breast | 39 | *BRCA2* | *c.6894C>G* | *p.Y2222** | Nonsense | Sister Breast (43), Mother Ovary (41), Brother Leukaemia (33), Mat Uncle Brain (40’s) | 20%  (BRCA1: 14%; BRCA2: 6%) |
| 868 | F | Chinese | Breast | 44 | *BRCA2* | *c.7878G>A* | *p.W2626** | Nonsense | Mat Aunt Cervix (65) | 11%  (BRCA: 4%;  BRCA2: 7%) |
| 86 | F | Malay | Breast | 43 | *BRCA2* | *c.9027T>G* | *p.Y3009** | Nonsense | Mother Peritoneum (70) | 15%  (BRCA1: 9%;  BRCA2: 6%) |
| 359 | F | Chinese | Breast | 38 | *BRCA2* | *c.9376C>T* | *p.Q3126** | Nonsense | Sister Ovary (53), Mother Brain (72), Mat Cousin Biliary (45), Mat Cousin Pancreas (40’s), Father Lung (72), Pat Aunt Breast (60’s), Pat Aunt Ovary (50’s) | 32%  (BRCA1: 26%; BRCA2: 6%) |
| 360  (Related to 359) | F | Chinese | Ovary | 53 | *BRCA2* | *c.9376C>T* | *p.Q3126** | Nonsense | Sister Breast (38), Mother Brain (72), Mat Cousin Biliary (45), Mat Cousin Pancreas (40’s), Father Lung (72), Pat Aunt Breast (60’s), Pat Aunt Ovary (50’s) | 32%  (BRCA1: 26%; BRCA2: 6%) |
| 1377 | F | Chinese | Breast | 51 | *BRCA2* | *c.9097insA* | *p.Thr3033Nfs* | FS Insertion | Brother Colon (62) / RCC (63), Sister Colon (61), Sister Bilateral Breast (40, 44), Sister Breast (51), Pat Uncle Gallbladder 951) / Lung (62), Pat Aunt Breast (85) | 13%  (BRCA1: 5%;  BRCA2: 8%) |
| 254 | F | Chinese | Breast | 36 | *BRCA2* | Duplication Exon 4-11 | Duplication | Duplication | Pat Uncle Gastric, Pat Cousin Ovary (45), Pat Cousin Breast (61), Pat Cousin AML | 27%  (BRCA1: 20%; BRCA2: 7%) |
| **919** | **F** | **South American** | **Breast** | **46** | ***BRCA2*** | ***c.3187_3188insG*** | ***p.Q1063Rfs*4*** | **FS Insertion** | **Sister Breast (53), Father Liver, Mat Uncle Brain** | **9%**  **(BRCA1: 3%;**  **BRCA2: 6%)** |

^†^ Novel mutations are underlined and in bold.

^‡^ The missense mutation in *RET* was re-confirmed on repeated testing in an independent laboratory.

Abbreviations: AML, acute myeloid leukemia; CA, cancer; FH, family history; FS, frameshift; HCC, hepatocellular carcinoma; Mat, maternal; NPC, nasopharyngeal carcinoma; Pat, paternal; RCC, renal cell carcinoma

**S3C Table. Pathogenic mutations in non-*BRCA1/2* genes and associated pedigree information (N=32)**

| **ID** | **Gender** | **Race** | **CA Primary** | **Age at diagnosis** | **Gene Affected** | **Nucleotide Change^1^** | **Amino-acid Change** | **Type of Mutation** | **Family CA History** | **PENN II Score** |
| --- | --- | --- | --- | --- | --- | --- | --- | --- | --- | --- |
| **331** | **F** | **Indian** | **Breast** | **64** | **ATM**  **ATM** | **c. 785T>A**  **c.8494C>T** | **p.L262***  **p.R2832C** | **Nonsense**  **Missense** | **Bro Bone Marrow (50s), Mat Uncle Liver (70), Mat Cousin Leukaemia (50), Mat Cousin Unknown (47), Mat Cousin Kidney (50), Mat Cousin Lung (59)** | **4%**  **(BRCA1: 1%;**  **BRCA2: 3%)** |
| 334 | F | Middle Eastern | Breast | 58 | BRIP1 | c.2947dupA | p.I983Nfs*19 | FS Duplication | Sister Brain (39), Half-brother Eye (4), Mother Endometrium (40’s), Mat Uncle Lung | 5%  (BRCA1: 1%;  BRCA2: 4%) |
| **762** | **F** | **Chinese** | **Ovary** | **62** | **BRIP1** | **c.505_506insAluY** | **p.Q169_Rfs*32** | **FS Insertion** | **No FH** | **NA** |
| 1291 | M | Caucasian | Pancreas | 57 | CDH1 | c.187C>T | p.R63* | Nonsense | Nephew Unknown, Mat Aunt Breast (50’s), Mat Greatgrandmother Pancreas, Pat Grandfather Colon (70’s) | 2%  (BRCA1: 0%;  BRCA2: 2%) |
| **1646** | **F** | **Caucasian** | **Breast** | **34** | **CHEK2** | **c.277delT** | **p.W93Gfs*17** | **FS Deletion** | **Mother AML (57), Father Plasmacytoma (61), Mat Grandmother Breast (50’s), Mat Grandaunt Breast, Pat Grandmother Gastric, Pat Grandfather Prostate, Pat Grandaunt Breast (60’s)** | **13%**  **(BRCA1: 6%;**  **BRCA2: 7%)** |
| **1479** | **F** | **Chinese** | **Breast** | **46** | **FANCC** | **c.339G>A** | **p.W113*** | **Nonsense** | **Pat Cousin Breast (29)** | **18%**  **(BRCA1: 10%; BRCA2: 8%)** |
| **926** | **F** | **Chinese** | **Ovary** | **64** | **FANCC**  BRCA1 | **c.1290C>A**  c.5525delT | **p.Y430***  p.V842Efs*131 | **Nonsense**  FS Deletion | **Sister Oral Cavity (42), Mother Colon / Endometrium (55)** | **NA** |
| 527 | F | Chinese | Ovary | 38 | MLH1 | c.1912G>T | p.G638* | Nonsense | Father Colon (60’s), Pat Uncle Colon (30’s), Pat Aunt Endometrium (50’s), Pat Aunt Endometrium (30’s), Pat Grandfather Colon (50’s) | NA |
| 1054 | F | Chinese | Breast | 45 | MLH1 | c.1946delC | p.P649Lfs | FS Deletion | Brother Colon (45), Father Colon (51), Sister Ovary (36), Pat Cousin Colon (50’s), Pat Cousin Colon / Ovary | 24%  (BRCA1: 19%; BRCA2: 5%) |
| 1074 | F | Chinese | Breast | 41 | MLH1 | c.2041G>A | p.A681T | Missense | Sister Colon (21), Father Oral Cavity (60) | 10%  (BRCA1: 4%;  BRCA2: 6%) |
| 1072 | F | Chinese | Ovary | 45 | MSH2 | c.942+3A>T | Deletion of Exon 5 | Intronic Change | Sister Endometrium (44), Sister Endometrium (41), Brother Colon (26), Brother Colon (50), Brother Colon (59), Mother Colon (57) / Kidney (75), Niece Colon (22) | NA |
| 59 | F | Chinese | Breast | 37 | *MUTYH*  *BRCA2* | *c.934-2A>G*  *c.774_775delAA* | *Splice acceptor in Intron 10*  *p.Q260Sfs*15* | FS Deletion  Splice Site Error | Mat Aunt Breast (50s), Pat Grandmother Throat (70s), Pat Cousin Breast, Pat Cousin Breast | 15%  (BRCA1: 8%;  BRCA2: 7%) |
| 838 | F | Chinese | Breast | 55 | MUTYH | c.934-2A>G | Splice Site Mutation | Splice Site Mutation | Sister NPC (30’s), Mat Uncle Colon (50’s), Mat Grandfather Gastric (60’s) | 9%  (BRCA: 5%;  BRCA2: 4%) |
| 893 | F | Chinese | Breast | 40 | MUTYH | c.934-2A>G | Splice Site Mutation | Splice Site Mutation | Pat Uncle Bladder / Prostate (70’s), Pat Uncle Lung (60’s), Pat Aunt Endometrium (60’s), Pat Aunt Breast (50’s), Pat Aunt Breast (50’s), Pat Cousin Leukaemia (19), Pat Grandmother Colon (60’s), Mat Cousin Breast (60), Mat Cousin Endometrium (60’s), Mat Grandmother Breast (60’s) | 11%  (BRCA1: 5%;  BRCA2: 6%) |
| 54 | F | Chinese | Breast | 39 | MUTYH | c.934-2A>G | Splice Site Mutation | Splice Site Mutation | Niece Wilm’s Tumour (3), Pat Aunt Breast (40’s) | 12%  (BRCA1: 6%;  BRCA2: 6%) |
| 1349 | F | Chinese | Breast | 32 | MUTYH | c.934-2A>G | Splice Site Mutation | Splice Site Mutation | Sister Breast (46), Mat Uncle Pancreas (68) | 27%  (BRCA1: 9%;  BRCA2: 18%) |
| 956 | F | Chinese | Ovary | 69 | MUTYH | c.934-2A>G | Splice Site Mutation | Splice Site Mutation | Sister Colon (70) | NA |
| 145 | F | Chinese | Ovary | 67 | MUTYH | c.934-2A>G | Splice Site Mutation | Splice Site Mutation | No FH | NA |
| **415** | **F** | **Middle Eastern** | **Breast** | **32** | **NBN** | **c.935T>A** | **p.L312*** | **Nonsense** | **Pat Aunt Endometrium (70’s), Pat Great Granduncle Gallbladder (77)** | **15%**  **(BRCA1: 7%;**  **BRCA2: 8%)** |
| **911** | **F** | **Chinese** | **Breast** | **30** | **PALB2** | **c.7G>T** | **p.E3*** | **Nonsense** | **Pat Uncle Unknown** | **16%**  **(BRCA1: 8%;**  **BRCA2: 8%)** |
| 753 | F | Chinese | Breast | 28 | PALB2 | Deletion of Exons 12-13 | Deletion | Large deletion | Brother Kidney (50), Mat Aunt Colon, Mat Aunt Breast, Mat Uncle Tongue, Mat Grandmother Bone, Mat Grandfather Lung | 17%  (BRCA1: 9%;  BRCA2: 8%) |
| **235** | **F** | **Chinese** | **Breast** | **50** | **RAD50** | **c.2165_2166insT** | **p.K722Nfs*6** | **FS Insertion** | **Sister Breast (64), Sister Breast (50’s), Sister Breast (50), Father Colon (70), Niece Endometrium (36),** | **8%**  **(BRCA1: 3%;**  **BRCA2: 5%)** |
| 236 | F | Chinese | Ovary | 53 | RAD51C | c.905-2A>C | Splice Site Mutation | Splice Site Mutation | Mother Liver / Lung (85) | NA |
| 1427 | F | Chinese | Ovary | 58 | RAD51D | c.270_271dupTA | p.K91Ifs*13 | FS Duplication | Sister Breast (40’s) | 13%  (BRCA1: 8%;  BRCA2: 5%) |
| 40 | F | Chinese | CA-free | NA | RET^‡^  BRCA2 | c.2410G.A^‡^  c.5645C>A | p.V804M^‡^  p.S1882* | Nonsense  Missense | Mother Breast (49), Mat Grandmother Bone Marrow (47), Mat Grandaunt Breast (50s), Mat Grandaunt Ovary (40s), Mat Granduncle Liver (60s), Mat Great Grandmother Unknowm, Pat Aunt Hodgkin’s (23) / Thyroid (39), Pat Grandfather Gastric (69), Pat Grandmother Pancreas (50) | 12%  (BRCA1: 7%;  BRCA2: 5%) |
| 648  (Related to 40) | F | Asian (Others) | Breast | 49 | RET^‡^  BRCA2 | c.2410G.A^‡^  c.5645C>A | p.V804M^‡^  p.S1882* | Nonsense  Missense | Mother Breast (47), Mat Aunt Breast (50s), Mat Aunt Ovary (40s), Mat Uncle Liver (60s), Mat Grandmother Unknown | 12%  (BRCA1: 7%;  BRCA2: 5%) |
| 1273 | F | Chinese | Adrenal | 29 | TP53 | c.329G>T | p.R110L | Missense | Pat Grandfather Skin (60’s), Pat Granduncle Lung (72), Mat Grandmother Thyroid (80), Mat Granduncle Colon (60’s) | 16%  (BRCA1: 8%;  BRCA2: 8%) |
| 1267 | F | Chinese | Ovary | 79 | TP53 | c.524G>A | p.R175H | Missense | No FH | NA |
| 137 | F | Chinese | Breast | 43 | TP53 | c.541C>T | p.R181C | Missense | Pat Uncle Intestine (70’s), Pat Uncle NPC (50’s), Pat Cousin Breast (30’s) | 14%  (BRCA1: 7%;  BRCA2: 7%) |
| **10** | **M** | **Indian** | **Colon** | **36** | **TP53** | **c.716delA** | **p.N239Tfs*8** | **FS Deletion** | **Mother Bilateral Breast (39, 47) / AML (51), Mat Aunt Breast (30’s)** | **7%**  **(BRCA1: 3%; BRCA2: 4%)** |
| 619 | M | Indian | Breast | 59 | TP53 | c.733G>A | p.G245S | Missense | Sister Colon (59), Mother Breast (75) / Colon (87) | 2%  (BRCA1: 1%;  BRCA2: 1%) |
| 1264 | F | Chinese | Breast | 24 | TP53 | c.743G>A | p.R248Q | Missense | Brother Adrenal (10), Pat Aunt Breast | 20%  (BRCA1: 11%; BRCA2: 9%) |

^1^ Novel mutations are underlined and in bold.

^‡^ The missense mutation in *RET* was re-confirmed on repeated testing in an independent laboratory.

Abbreviations: AML, acute myeloid leukemia; CA, cancer; FH, family history; FS, frameshift; HCC, hepatocellular carcinoma; Mat, maternal; NPC, nasopharyngeal carcinoma; Pat, paternal; RCC, renal cell carcinoma
